# Supplementary material for: Loss of the Immunomodulatory Transcription Factor BATF2 in Humans Is Associated with a Neurological Phenotype
Source: Cells. 2023 Jan 5;12(2):227. doi: 10.3390/cells12020227 (PMC9856319; doi:10.3390/cells12020227)
Supplement: Supplementary file 1 [file cells-12-00227-s001.zip › BATF2_Supplement_clean.pdf]

Supplementary Information

Loss of the immunomodulatory transcription factor BATF2 in humans is associated with a neurological phenotype

Gábor Zsurka, Maximilian Appel, Maximilian Nastaly, Kerstin Hallmann, Niels Hansen, Daniel Nass, Tobias Baumgartner, Rainer Surges, Gunther Hartmann, Eva Bartok, Wolfram S. Kunz

Table S1
Clinical and laboratory features of patients

Patient	P1	P2	P3
**Gender**	female	female	female
**Current age**	26	23	15
**Major clinical features at presentation**	mental retardation, behavioral disorder with aggression, epilepsy	mental retardation, behavioral disorder with aggression and epilepsy, hyperactivity	severe mental retardation, epilepsy
**Minor clinical features at presentation**	sleeping disorder	deformity of spine, sleeping disorder, gastroesophageal reflux	hyperflexible joints, pes equinus, small stature
**Epilepsy onset**	14	14	1.5
**Focal seizures**	yes (focal seizures with impaired awareness)	yes (focal seizures with impaired awareness)	yes (focal seizures with impaired awareness)
**Gelastic seizures**	yes	yes	no
**Tonic clonic seizures**	yes (focal to bilateral tonic-clonic seizures)	yes (focal to bilateral tonic-clonic seizures)	yes
**Tonic seizures**	no	no	yes
**Atonic seizures**	no	no	yes
**Current frequency of seizures per month**	2	none	10
**Status epilepticus (age of onset)**	yes (18)	no	yes (14)
**Febrile seizure (age of onset)**	yes (1)	no	no
**Psychomotor retardation onset**	3	2.7	1.5
**Neurological examination**	gait abnormality with outer rotation of hips and feet	gait abnormality, ataxia	gait abnormality with spastic tetraparesis and ataxia
**Psychopathological examination**	aggressive, dysphoric behavior, moderate cognitive impairment	aggressive, dysphoric behavior with echolalia, moderate cognitive impairment	severe cognitive impairment, friendly nature
**Endocrinological status**	normal	normal	precocious puberty
**Hematological status**	thrombocytopenia	thrombocytopenia, mild leukopenia	thrombocytopenia, severe leukopenia, anemia
**Immunstatus PB**	normal	complete lack of B-cells	low natural killer cells
**Infection status**	normal	normal	frequent gastroenteritis and recurrent herpes labialis
**Rheumatological and dermatological status**	Arthralgia	normal	normal
**Autoantibodies PB**	anti-ANA (1:320), ANA subtypes were not identified	borderline titre of anti-ANA (1:80)	no
**EEG**	alpha-EEG with diffuse ß-activity	alpha-EEG	diffuse slowing, multifocal generalized epileptiform discharges
**Cranial MRI**	no abnormalities	no abnormalities	no abnormalities
**CSF**	normal	normal	CSF protein elevated
**Prior AED treatment**	BRV, CBZ, LCM, LEV, LTG, ESL	CBZ, BRV, LCM, LTG, LEV	CBZ, LCM, LTG, LEV, PG
**Current AED treatment**	ZNS, VPA	VPA, ZNS	OXC, TPM, VPA, CLB

AED, antiepileptic drugs; ANA, antinuclear antibodies; BRV, brivaracetam; CBZ, carbamazepine; CLB, clobazam; ESL, eslicarbazepinacetat; LCM, lacosamide; LEV, levetiracetam; LTG, lamotrigine; OXC, oxcarbazepine; PB, peripheral blood; PG, pregabalin; TPM, topiramate; VPA, valproic acid

Table S2

Common runs of homozygosity (ROH) and a single common homozygous deletion in the three patients

Run of homozygosity	ROH size	Deletion	Deletion size	Affected genes
chr2:198,085,516-202,074,931	3,989,416			
chr7:130,383,698-136,903,096	6,519,399			
chr11:46,434,794-56,319,084	9,884,291	chr11: 55,603,479-55,651,839	48,361	*OR4C11, OR4P4, OR4S2*
chr11:62,391,452-66,643,313	4,251,862			
chr11:67,289,638-71,789,537	4,499,900			
chr12:70,684,355-70,920,322	235,968			
chr17:1,083,502-2,962,525	1,879,024			
chr19:10,707,416-16,019,784	5,312,369			

Table S3

Rare homozygous variants detected in the three patients

Gene name	Chromosomal position[a]	Transcript ID	Transcript change	Amino acid change	CADD score	Allele frequency in gnomAD	Parents' genotypes (mother; father)[b]
BATF2	11:64994534G>A	NM_138456.3	c.55C>T	p.Gln19*	35	0	het; het
UNC93B1	11:67999231C>T	NM_030930.3	c.629G>A	p.Arg210Gln	22.8	5	het; het
EPOR	19:11378451G>C	NM_000121.3	c.1060C>G	p.Pro354Ala	19.1	0	het; hom
ZNF709	19:12466733C>T	NM_152601.3	c.121G>A	p.Ala41Thr	19.5	0	het; hom
CCDC105	19:15011213G>T	NM_173482.2	c.388G>T	p.Ala130Ser	22.9	45 (1 hom)	het; hom

[a]Chromosomal positions are indicated according to human genome assembly GRCh38.p12.

[b]het, heterozygous; hom, homozygous.

Figure S1

Knockout THP-1 cell lines generated using CRISPR–Cas9

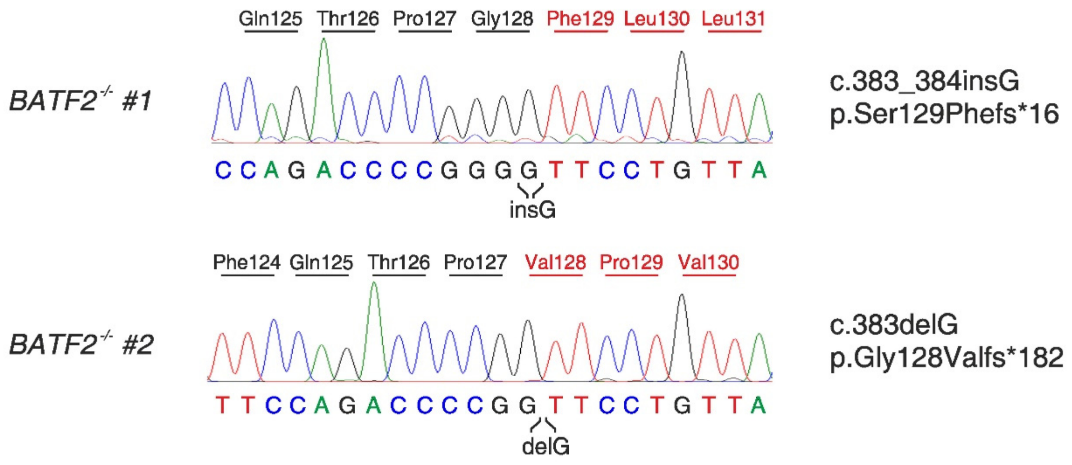

Sequencing chromatograms confirming knockout genotypes of *BATF2*. Red amino acid codes indicate missense changes downstream of frame shifts.

Figure S2

Differentially expressed genes in non-stimulated *BATF2*$^{-/-}$ and wild-type THP-1 cells as determined by 3' RNA sequencing

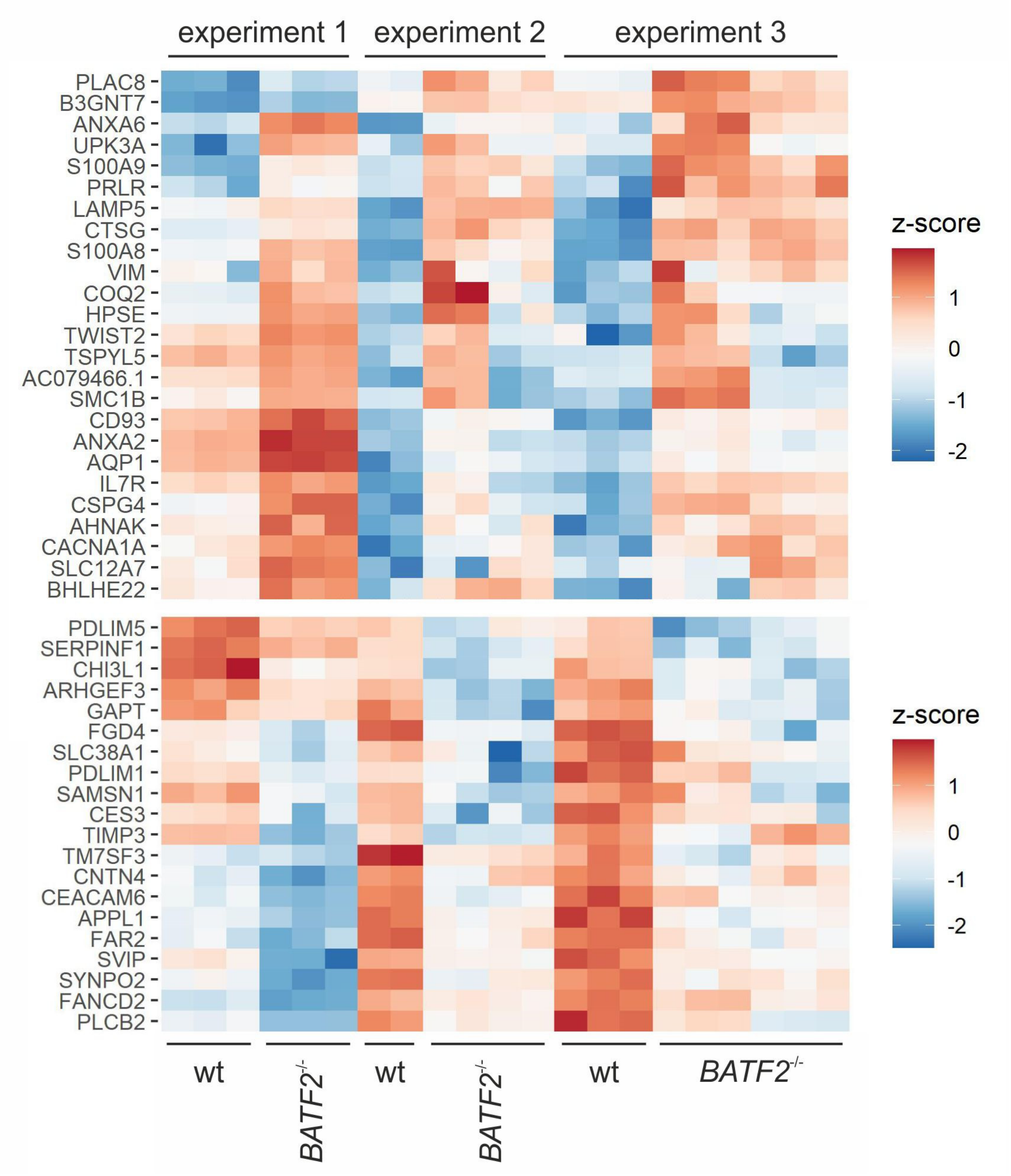

Heatmaps of z-scores of normalized gene counts. Genes are listed that show significant upregulation (upper panel) or downregulation (lower panel) in *BATF2*$^{-/-}$ THP-1 cells (FDR < 0.05) as compared to wild-type cells in all three independent experiment. Gene ontology pathway enrichment analysis does not return any significant hit.

Legend to Supplemental Video

Video-EEG monitoring of patient P3. EEG: In the forerun (not shown in the video) non-rapid eye movement sleep (NREM, stage III). Initial generalized EEG suppression, followed by rhythmic theta activity predominantly right fronto-temporal. Semiology: Awakening, Staring, oral automatisms and mild hyperkinetic movements. Evaluation: Focal motor seizure. Awareness was not tested.
